# Waist-to-height ratio associated cardiometabolic risk phenotype in children with overweight/obesity

**DOI:** 10.1186/s12889-023-16418-9

**Published:** 2023-08-15

**Authors:** Tochi E. Ukegbu, Judith Wylie-Rosett, Adriana E. Groisman-Perelstein, Pamela M. Diamantis, Jessica Rieder, Mindy Ginsberg, Alice H. Lichtenstein, Nirupa R. Matthan, Viswanathan Shankar

**Affiliations:** 1 Sophie Davis School of Biomedical Education, The City College of New York, 160 Convent Ave, New York, NY 10031 USA; 2 Department of Epidemiology and Population Health, Albert Einstein College of Medicine, 1300 Morris Park Avenue, Bronx, NY 10461 USA; 3 Department of Pediatrics, Albert Einstein College of Medicine, Jacobi Medical Center, NY 10461 Pelham Pkwy S, Bronx, USA; 4 Department of Pediatrics, Albert Einstein College of Medicine Children’s Hospital at Montefiore, Bronx, NY 10467 USA; 5 Cardiovascular Nutrition Laboratory, JM USDA Human Nutrition Research Center on Aging, Tufts University, 711 Washington St, MA 02111 Boston, USA

**Keywords:** Cardiometabolic risk, Waist-to-height ratio, Insulin resistance, Liver enzymes, AST, ALT

## Abstract

**Background:**

Childhood overweight/obesity has been associated with an elevated risk of insulin resistance and cardiometabolic disorders. Waist-to-height ratio (WHtR) may be a simple screening tool to quickly identify children at elevated risk for cardiometabolic disorders. The primary objective of the present study was to create sex-specific tertile cut points of WHtR and assess its association with Insulin resistance and elevated liver enzyme concentrations in children, factors using cross-sectional data from the randomized, controlled Family Weight Management Study.

**Methods:**

Baseline data from 360 children (7–12 years, mean Body Mass Index (BMI) ≥ 85^th^ percentile for age and sex) were used to calculate WHtR tertiles by sex, male: ≤ 0.55 (T1), > 0.55- ≤ 0.59 (T2), > 0.59 (T3); female: ≤ 0.56 (T1), > 0.56- ≤ 0.6 (T2), > 0.6 (T3). The Homeostatic Model Assessment for Insulin Resistance (HOMA-IR) was used to categorize participants as insulin-resistant (HOMA-IR ≥ 2.6) and insulin-sensitive (HOMA-IR < 2.6). Liver enzymes aspartate aminotransferase (AST) and alanine aminotransferase (ALT) were categorized as normal vs. elevated (AST of < 36.0 µkat/L or ≥ 36.0 µkat/L; ALT of < 30.0 µkat/L or ≥ 30.0 µkat/L; ALT > 26 µkat/L males, > 22 µkat/L females). We examined differences in baseline cardiometabolic risk factors by WHtR tertiles and sex-specific multivariable logistic regression models to predict HOMA-IR and elevation of liver enzymes.

**Results:**

Study participants had a mean WHtR of 0.59 ([SD: 0.06]). Irrespective of sex, children in WHtR T3 had higher BMIz scores, blood pressure, triglycerides, 2-h glucose, fasting 2-h insulin, and lower high-density lipoprotein cholesterol (HDL-C) concentrations than those in T2 and T1. After adjusting for covariates, the odds of elevated HOMA-IR (> 2.6) were over five-fold higher among males in T3 versus T1 [OR, 95%CI: 5.83, 2.34–14.52] and T2 [OR, 95%CI: 4.81, 1.94–11.92] and females in T3 [OR, 95%CI: 5.06, 2.10–12.20] versus T1. The odds of elevated ALT values (≥ 30) were 2.9 [95%CI: 1.01–8.41] fold higher among females in T3 compared to T1.

**Conclusion:**

In public health settings, WHtR may be a practical screening tool in pediatric populations to identify children at risk of metabolic syndrome.

**Supplementary Information:**

The online version contains supplementary material available at 10.1186/s12889-023-16418-9.

## Background

Obesity, characterized by the excessive accumulation of adipose tissue, increases cardiometabolic risk factors such as hyperglycemia, dyslipidemia, and insulin resistance (IR), has increased dramatically over the past few decades [1, 2]. Many of these adverse metabolic factors are strongly associated with their prevalence later in life. Although various anthropometric and biochemical measures are viable markers for cardiometabolic risk detection in adults, there is a lack of substantial research examining the accuracy of these measures as predictors in children [3]. Childhood obesity is also associated with an increased risk of developing non-alcoholic fatty liver disease (NAFLD) later in life, with one of the histological stages being liver fibrosis [4]. Hepatic ectopic lipid deposition can result in inflammation and liver fibrosis [5–7]. Obesity and IR increase liver fibrosis risk [8, 9]. Measures of liver function, such as aspartate aminotransferase (AST) and alanine aminotransferase (ALT), are indicators of cellular liver injury [10]. The lack of research on the pathogenesis of liver fibrosis in children and the unavailability of current assessment methods further emphasizes the need for simple, noninvasive methods to assess this condition [11, 12]. 

Waist-to-height ratio (WHtR), derived as a ratio of waist circumference in centimeters (cms) by height (cms), a simple screening tool [13], has recently been proposed as a diagnostic measure of early cardiometabolic risk in both children [3, 14–16] and adults [16–19]. WHtR is more strongly associated with cardiovascular and cardiometabolic risk factors than individual anthropometric measures, such as waist circumference, Body Mass Index (BMI), and waist-hip ratio [15, 17, 20–24]. [21, 22]. The results of systematic reviews and meta-analyses of WHtR in adults have suggested values above 0.5 represents increased cardiometabolic risk [14, 25]. A recent meta-analysis [18] examining WHtR values in children and adolescents suggested a single cutpoint of 0.49 for both boys and girls. In contrast, a second meta-analysis [16] did not support this conclusion.

The aim of the study was to establish sex-specific tertile cut points of WHtR and assess their association with Insulin Resistance (IR), cardiometabolic risk factors, and elevated liver enzyme concentrations in children 7–12 years. Our analyses explore the potential to identify the risk phenotype using the waist-to-height ratio (WHtR) [25].

## Methods

### Setting

The study utilized baseline data from the Family Weight Management Study (also known as the Fun Healthy Families study), a randomized controlled trial [26] conducted from 2009 to 2013 in a pediatric ambulatory program of an urban hospital that provides safety-net primary care services in the Bronx, New York, United States.

### Participants

Study participants (*N* = 360) included children aged 7–12 years with a BMI ≥ 85th percentile for age and sex [27]. Exclusion criteria for the participants included any chronic illnesses, a physical, cognitive, or emotional impairment that would impact the safety of participants during study procedures, medical treatment causing fluctuations in body weight, inconvenient transportation distances, involvement in a separate weight management program, and unwillingness or inability of the parents or child to provide consent and assent, respectively. The trial design with the CONSORT diagram and study process is described elsewhere [26]. The Albert Einstein College of Medicine Institutional Review Board (IRB) approved all study protocols; all study participants provided written consent (parent or guardian) or assent (children).

### Anthropometric measures

Height and weight were measured in light clothing and without shoes. A stadiometer and a digital scale were used to obtain height and weight, respectively. The waist circumference was measured using an elastic tape at the iliac crest, and the hip circumference at the point of maximal protrusion of the gluteal muscles in the lateral position. Both were recorded to the nearest centimeter. Scales and stadiometer were calibrated, and anthropometry tapes were examined for signs of wear weekly using standardized protocols.

### Cardiometabolic parameters

As previously reported in Wylie-Rosett et al. [26], systolic and diastolic blood pressures were measured three times according to traditional pediatric standards using appropriate cuff size with a manual sphygmomanometer after sitting for 2 min. Blood specimens were obtained after a minimum of an 8-h fast. Fasting glucose, triglyceride (TG), total cholesterol (TC), low-density lipoprotein (LDL) cholesterol, and high-density lipoprotein (HDL) cholesterol concentrations were measured spectrophotometrically using a Beckman-Coulter LX-20 auto-analyzer (Brea, CA). A glucose amount of 1.75 g/kg body weight (GlucolaTM) was administered for the 2-h Oral Glucose Tolerance Test. The liver enzymes, alanine transaminase (ALT/SGPT) and aspartate aminotransferase (AST/SGOT), concentrations were measured using an Immulite 2000 analyzer (Bio-DPC; Siemens Medical, Gwynedd, UK).

### Intermediate parameters

The following variables were used as markers for increased cardiometabolic risk:

- WHtR parameters: There is no consensus on the appropriate cut points of WHtR in pediatric populations [15–18, 20, 28]. Previous reviews and analyses have indicated that dichotomized WHtR cut points at ≥ 0.5 [18] or 0.55 [29] are surrogates of increased risk in children; however, they may be insignificant when assessed for sensitivity and specificity to certain variables [30–32]. Therefore, the following cut points by sex were used based on the sample data to group children into three categories: females WHtR ≤ 0.56 (T1), WHtR > 0.56—≤ 0.60 (T2), and WHtR > 0.60 (T3); males WHtR ≤ 0.55 (T1), WHtR > 0.55—≤ 0.59 (T2), and WHtR > 0.59 (T3).
- Insulin Resistance (IR): IR is a critical component of cardiovascular disease and metabolic syndrome (MetS) [1, 33]. Though increased HOMA-IR values are associated with higher risk, no clear cut point is used to assess IR in pediatric clinical studies [33]. We used HOMA-IR values < 2.6 and ≥ 2.6 to evaluate increased cardiometabolic risk based on prior published research from the Family Weight Management Study [34, 35].
- Liver enzymes: Measures of serum AST(SGOT) and ALT(SGPT) levels have been used extensively in studies to assess liver damage [10]. There are different cut points proposed [36–38]. AST values of < 36.0 µkat/L and ≥ 36.0 µkat/L and ALT values of < 30.0 µkat/L and ≥ 30.0 µkat/L are proposed as cut points associated with increased risk of liver injury in children [39] and adopted for this study. In addition to these cut points, we also examined the following cut points 22 µkat/L for girls and 26 µkat/L for boys based on the North American Society For Pediatric Gastroenterology, Hepatology & Nutrition (NASPGHAN) Clinical Practice Guideline review [36, 38].

### Statistical analysis

A sex-specific demographic, anthropometric, and cardiometabolic biomarker distribution was summarized using descriptive statistics. Normally distributed continuous variables were numerically summarized using mean (standard deviation), while non-normally distributed were presented with median (interquartile range). The categorical variables were presented as frequency counts and percentages. The difference in child characteristics among the WHtR tertile categories (sex-specific) was assessed using analysis of variance, the Kruskal–Wallis test, or the Pearson chi-square test. We modeled HOMA-IR, AST, and ALT values as binary variables for association models. The association between WHtR tertile categories (sex-specific) and outcome variables HOMA-IR, AST, and ALT, adjusting for the other covariates, was examined using a multivariable logistic regression model. Firth's bias-corrected logistic regression was used to associate WHtR and outcomes with a small number of events or when the issue of quasi or complete separation arose. We also modeled binary WHtR cut points (> 0.5 v ≤ 0.5; > 0.55 v ≤ 0.55; M: > 0.59 v ≤ 0.59, F: > 0.60 v ≤ 0.60) for comparison. Covariates (child’s age, race, ethnicity, household income, parent’s education, occupation, Tanner stage) that were significantly different at the 20% level at the univariable model as were demographic confounders were considered for the multivariable model. Final multivariable models were adjusted for the child’s age, race, ethnicity, parent’s education, and occupation; in addition, HOMA-IR models were adjusted for the tanner stage. The Tanner stage variable had 13.6% missing data, which was addressed using a fully conditional specification multiple imputations approach. Ten imputation data sets were generated, and estimates were pooled using Rubin’s rules [40].

## Results

### Participant Characteristics

Three hundred and sixty children participated in the study, of which 52% (*n* = 185) were females and 48% (*n* = 175) were males. Seventy-four percent (*n* = 267) self-identified as Hispanic, 17.5% (*n* = 63) as non-Hispanic African American or Black, and 8.3% as non-Hispanic origin, others including Caucasian or White, Asian, Hawaiian, and multiracial. The average age of children was 9.3 (SD: 1.7). A detailed summary of participant characteristics has been previously reported [26]. The average WHtR among the participants was 0.59 (SD: 0.06). The average HOMA-IR, AST(SGOT), and ALT (SGPT) values were 3.68(SD: 2.58), 25.83 (SD: 17.57), and 30.96(SD: 9.40), respectively (Table 1). The demographic, anthropometric, and cardiometabolic characteristics distribution between the sex-specific WHtR tertile were similar to different WHtR categories. For comparisons with our proposed WHtR tertile categories, we also categorized WHtR by commonly used cut points and their distribution by sex is presented in Supplemental Table 1

**Table 1:** Distribution of participant demographic, anthropometric, and cardiometabolic biomarkers by sex-specific waist-to-height ratio tertile categories

Variable	Waist-to-Height Ratio							
	**Male (*n* = 175)**				**Female (*n* = 185)**			
	**T1 (*n* = 58) ≤ 0.55**	**T2 (*n* = 59) > 0.55- ≤ 0.59**	**T3 (*n* = 58) > 0.59**	***P*-value**	**T1 (*n* = 61) ≤ 0.56**	**T2 (*n* = 62) > 0.56- ≤ 0.6**	**T3 (*n* = 62) > 0.6**	***P* Value**^**†**^
**Age (years)**^**a**^	9.2 (1.8)	9.2 (1.7)	9.3 (1.6)	0.95	9.0 (1.8)	9.5 (1.8)	9. 5 (1.7)	0.19
**Race/ethnicity n (%)**								
Hispanic	45 (77.6)	44 (74.6)	43 (74.1)		44 (72.1)	47 (75.8)	44 (71.0)	0.78^§^
Non-Hispanic AA	8 (13.8)	11 (18.6)	10 (17.2)	0.96^§^	11 (18.0)	9 (14.5)	14 (22.6)	
Non-Hispanic White & Others	5 (8.6)	4 (6.8)	5 (8.6)		6 (9.8)	6 (9.7)	4 (6.5)	
**Parent education n (%)**								
< High School	30 (51.7)	27 (45.8)	28 (48.3)	0.83^§^	29 (47.5)	29 (46.8)	32 (51.6)	0.39^§^
High school or GED	13 (22.4)	17 (28.8)	18 (31.0)		15 (24.6)	21 (33.9)	12 (19.4)	
> High school	15 (25.9)	15 (25.4)	12 (20.7)		17 (27.9)	12 (19.4)	18 (29.0)	
**Parent occupation n (%)**								
Employed full time	9 (15.5)	10 (17.0)	10 (17.2)	1.00^§^	11 (18.0)	13 (21.0)	16 (25.8)	0.46^§^
Employed part-time	10 (17.2)	9 (15.3)	9 (15.5)		7 (11.5)	13 (21.0)	9 (14.5)	
Other (retired Homemaker unemployed)	39 (67.2)	40 (67.8)	39 (67.2)		43 (70.5)	36 (58.1)	37 (59.7)	
**Height (cm) **^**a**^	140.6 (12.2)	139.2 (10.6)	141.8 (11.7)	0.50	138.6 (11.9)	142.2 (11.4)	142.2(11.5)	0.14
**Weight (lbs) **^**a**^	98.4 (23.9)	107.5 (26.1)	131.5 (35.0)	** < .0001**	95.5 (23.1)	113.1 (28.3)	134.0 (37.4)	** < .0001**
**BMI Z score**^**a**^	1.7 (0.29)	2.1 (0.3)	2.4 (0.2)	** < .0001**	1.6 (0.3)	1.9 (0.3)	2.3 (0.2)	** < .0001**
**SBP (mmHg) **^**a**^	104.0 (7.5)	106.6 (9.3)	111.4 (11.8)	**0.0002**	102.2 (8.8)	106.3 (10.7)	109.5 (12.3)	**0.001**
**DBP (mmHg)**^**a**^	57.4 (5.2)	57. 7 (5.3)	59.8 (6.1)	**0.05**^**¥**^	57.1 (4.8)	58.5 (6.1)	60.0 (5.6)	**0.02**
**Triglycerides (mg/dL)**^**b**^	63 (47–90)	73(49–101)	72(59–98)	0.16^**¥**^	71 (52–113)	79(60–113)	86(61–126)	**0.07**^**¥**^
**Total cholesterol (mg/dL)**^**a**^	154.5 (27. 7)	153.5 (29.2)	159.5 (26.1)	0.46	157.6 (26.9)	151.3 (30.6)	158.1 (29. 4)	0.35
**HDL-C (mg/dL)**^**a**^	49.9 (10.2)	46.6 (7.9)	45.4 (9.2)	**0.03**	48.1 (9.8)	44.3 (9.0)	43.3 (9.7)	**0.01**
**LDL-C (mg/dL)**^**a**^	90.5 (23.1)	91.4 (25.1)	97.0 (20.4)	0.26	93.3 (23.2)	91.2 (20.7)	93.6 (25.5)	0.83
**Fasting glucose (mg/dL)**^**a**^	84.6 (7.1)	86.2 (7.1)	86.0 (10.5)	0.55	84.0 (8.1)	84. 6 (13.4)	84.6 (7.4)	0.93
**Glucose 2 HR (mg/dL)**^**a**^	93.3 (13. 7)	96.7 (15.0)	105. 8 (18.6)	**0.0001**	92.30 (16.4)	95.2 (20. 0)	100.9 (17.5)	**0.03**
**Fasting insulin (μU/mL)**^**b**^	10.3 (7.0–15.5)	12.5(9.9–18.2)	16.5 (11.6–23.4)	**0.0001**^**¥**^	11.8 (8.2–16.5)	18.0 (11.5–28.0)	18.2(13.0–27.9)	** < .0001**^**¥**^
**Fasting Insulin 2 HR (μU/mL)**^**b**^	49.3 (26.3–72.1)	47.0 (27.5–74.1)	76.7(49.2–135.0)	**0.0003**^**¥**^	46.9 (34.3–77.5)	92.9(63.8–157.1)	99.9(44.7–204.1)	** < .0001**^**¥**^
**HOMA-IR**^**b**^	2.0 (1.40–3.37)	2.5 (2.2–4.0)	3.5(2.3–5.3)	**0.0002**^**¥**^	2.3 (1.5–3.4)	3.8 (2.5–5.9)	3.9 (2.6–6.1)	** < .0001**^**¥**^
**ALT (SGPT) (µkat/L)**	21 (19–28)	24 (19–29)	27(20–35)	**0.03**^**¥**^	20 (17–24)	22(18–25)	22(18–29)	0.08^**¥**^
**AST (SGOT) (μkat/L)**^**b**^	31 (26–35)	32(27–35)	32(26–36)	0.82^**¥**^	30 (27–34)	27(25–33)	28(25–32)	0.10^**¥**^
**Tanner Stage**^**b**^	1 (1–2)	1 (1–2)	1 (1–2)	0.84^**¥**^	1(1–2)	2 (1–2)	2(1–2)	0.06^**¥**^

^†^Analysis of Variance

^§^Pearson Chi-square test

¥Kruskal Wallis Test

^a^ values are mean (SD)

^b^values are median (IQR)

### Differences in cardiometabolic risk parameters

In both sexes, cardiometabolic risk markers, including BMI- z score, SBP, DBP, and 2-h glucose, fasting, and 2-h insulin concentrations, were lowest in children in WHtR T1, intermediate in WHtR T2 and highest in WHtR T3 among categories (Table 1), with markers showing linear relation (either a monotonic increase or decrease) among the WHtR tertile categories in both sexes. HDL-cholesterol concentration was lowest in the WHtR T3 category in both males and females, consistent with the previously reported observation of an inverse relationship between adiposity and HDL-cholesterol concentrations [41]. The liver function biomarker (SGPT/ALT) was positively associated with concentrations that were higher with increasing WHtR categories in males (*p* = 0.03) but did not reach statistical significance in females (*p* = 0.08).

### Association of WHtR with insulin resistance and liver biomarkers

The complete case and multiple imputation model estimated for the HOMA-IR are presented in Table 2. Based on a multiple imputation analysis, female children in T3 (WHtR > 0.60) had a 5.06 (95% CI: 2.10–12.20) fold higher odds of being insulin resistant (HOMA-IR > 2.6) than those in T1 (WHtR ≤ 0.56) (Table 2). The odds of insulin resistance were 4.81 (95%CI: 1.94–11.92) fold higher among T2 than T1. Similarly, the odds of insulin resistance were 5.83 (95%CI: 2.34–14.52) fold higher among T3 (WHtR > 0.59) category than T1 (WHtR ≤ 0.55) among the males. The effect size was not statistically significant and was half for those in T2. We also compared established WHtR binary cut points of > 0.5 [18], > 0.55 [29] and > 0.6 [25, 42, 43]. The gender-specific adjusted OR for binary cut points (males > 0.59 and females > 0.60) were 4.54 (95%CI: 2.17–9.50) and 2.54 (95%CI: 1.22–5.26) for males and females, respectively. The established conservative binary cut points showed elevated risk, but the strength of association was smaller.

**Table 2:** Odds Ratio, 95% confidence interval, and P-value from multivariable logistic regression for HOMA-IR

Variable		Male			Female		
		**aOR**	**95% CI**	***P*-value**	**aOR**	**95% CI**	***P*-value**
**WHtR**^**#**^	T1 (ref)	1			1		
	T2	1.66	0.66–4.17	0.2783	**5.38**	**2.00–14.49**	**0.0009**
	T3	**5.56**	**2.02–15.28**	**0.0009**	**5.68**	**2.23–14.48**	**0.0003**
**WHtR**^#^*	T1 (ref)	**1**			1		
	T2	2.00	0.83–4.83	0.1218	**4.81**	**1.94–11.92**	**0.0007**
	T3	**5.83**	**2.34–14.52**	**0.0002**	**5.06**	**2.10–12.20**	**0.0003**
**WHtR**^**§**^	M: ≤ 0.59; F: ≤ 0.60 (ref)	1			1		
	M: > 0.59; F: > 0.60	**4.51**	**1.99–10.22**	**0.0003**	**2.31**	**1.09–4.92**	**0.0299**
**WHtR**^§*^	M: ≤ 0.59; F: ≤ 0.60 (ref)	**1**			**1**		
	M: > 0.59; F: > 0.60	**4.54**	**2.17–9.50**	** < 0.0001**	**2.54**	**1.22–5.26**	**0.0124**
**WHtR**	≤ 0.55 (ref)	**1**			1		
	> 0.55	**3.26**	**1.38–7.69**	**0.0072**	**3.73**	**1.54–9.03**	**0.0035**
WHtR^*^	≤ 0.55 (ref)	1			**1**		
	> 0.55	**3.99**	**1.73–9.17**	**0.0011**	**3.31**	**1.49–7.37**	**0.0033**
**WHtR**	≤ 0.5 (ref)	1			1		
	> 0.5	1.16	0.21–6.46	0.8673	2.63	0.43–16.08	0.2944
WHtR^*^	≤ 0.5 (ref)	1	0.21–6.46	0.8673	2.63	0.43–16.08	0.2944
	> 0.5	2.00	0.38–10.58	0.4151	2.77	0.47–16.41	0.2603

^#^ waist-to-height ratio tertiles: Male: ≤ 0.55 T1, > 0.55- ≤ 0.59 T2, > 0.59 T3; Female: ≤ 0.56 T1, > 0.56- ≤ 0.6 T2, > 0.6 T3; (ref)-reference category

*multiple imputations

§ Male ≤ 0.59 v > 0.59, Female ≤ 0.6 v > 0.6, & ≤ 0.50 v > 0.50

All models adjusted for age, race/ethnicity, parents education, occupation, and Tanner stage

ALT as a marker for liver injury in children was assessed using two cut-off criteria: (i) ≥ 30 vs. < 30) and NASPGHAN sex-specific criteria of > 26 vs. ≤ 26 for males and > 22 vs. ≤ 22 for females. Among the females, the odds of elevated ALT (≥ 30) were 2.9-fold higher among T3 compared to the T1 WHtR category (aOR, 2.92; 95% CI: 1.01, 8.41). Although an elevated association was observed in the T2 WHtR category (aOR = 1.77; 95%CI: 0.59, 5.35), the difference did not reach statistical significance (Table 3). Among males, there was a non-significant elevated association between T3 WHtR (aOR = 1.87) and T2 WHtR (aOR = 1.13). When assessed using the NASPGHAN criteria, among both female and male children, the odds of elevated ALT (males > 26; female > 22) showed stronger associations in theT3 WHtR than the T1 category. A non-significant positive association was observed between AST and WHtR among males but not females (Table 4).

**Table 3:** Odds Ratio, 95% confidence interval, and P-value from multivariable logistic regression for ALT/SGPT

Variable		Male			Female		
		**aOR**	**95% CI**	***P*-value**	**aOR**	**95% CI**	***P*-value**
**ALT/SGPT: < 30, ≥ 30**							
**WHtR**^**a**^	T1 (ref)	1			1		
	T2	1.13	0.46–2.80	0.7847	1.77^c^	0.59–5.35	0.3128
	T3	1.87	0.79–4.40	0.1550	**2.92**^c^	**1.01–8.41**	**0.0471**
**WHtR**^**b**^	M: ≤ 0.59; F: ≤ 0.60 (ref)	1			**1**		
	M: > 0.59; F: > 0.60	1.48	0.73–3.00	0.2850	**2.44**^c^	**1.05–5.66**	**0.0373**
**WHtR**	≤ 0.55 (ref)	1					
	> 0.55	1.48	0.67–3.27	0.3340	**1**		
	≤ 0.5 (ref)				**3.06**^**c**^	**0.89–8.31**	**0.0803**
**WHtR**	> 0.5	1	0.30–22.37	0.3849	1		
		2.60			3.92^c^	0.16–96.5	0.4033
**ALT/SGPT: Male: ≤ 26, > 26 / Female: ≤ 22, > 22**							
**WHtR**^**a**^	T1 (ref)	1			1		
	T2	1.69	0.75–3.84	0.2076	1.84	0.85–3.96	0.1217
	T3	**3.17**	**1.41–7.13**	**0.0052**	**2.23**	**1.04–4.82**	**0.0405**
**WHtR**^**b**^	M: ≤ 0.59; F: ≤ 0.60 (ref)	1					
	M: > 0.59; F: > 0.60	**2.28**	**1.19–4.40**	**0.0134**	**1.83**	**0.98–3.42**	**0.0592**
**WHtR**	≤ 0.55 (ref)	1			**1**		
	> 0.55	**2.24**	**1.08–4.67**	**0.0308**	**1.96**	**0.93–4.10**	**0.0759**
**WHtR**	≤ 0.5 (ref)	1			1		
	> 0.5	2.08	0.39–11.15	0.3908	5.40	0.62–46.99	0.1270

^a^ waist-to-height ratio tertiles: Male: ≤ 0.55 T1, > 0.55- ≤ 0.59 T2, > 0.59 T3; Female: ≤ 0.56 T1, > 0.56- ≤ 0.6 T2, > 0.6 T3; (ref)-reference category

^b^ Male ≤ 0.59 v > 0.59, Female ≤ 0.6 v > 0.6, & ≤ 0.50 v > 0.50

all models adjusted for age, race/ethnicity, parents education, occupation

^c^ Firth Bias Corrected Logistic regression

**Table 4:** Odds Ratio, 95% confidence interval, and P-value from multivariable logistic regression for AST/SGOT (< 36, ≥ 36)

Variable		Male			Female		
		**aOR**	**95% CI**	***P*-value**	**aOR**	**95% CI**	***P*-value**
**WHtR**^**a**^	T1 (ref)	1			1		
	T2	1.10	0.44–2.70	0.8440	1.40^c^	0.48–4.06	0.5388
	T3	1.83	0.77–4.36	0.1753	1.03^c^	0.35–3.02	0.9595
**WHtR**^**b**^	M: ≤ 0.59; F: ≤ 0.60 (ref)	1					
	M: > 0.59; F: > 0.60	1.47	0.71–3.05	0.2985	1.11^c^	0.45–2.71	0.8241
**WHtR**	≤ 0.55 (ref)	1			1	0.36–2.59	0.9541
	> 0.55	1.49	0.67–3.29	0.3236	0.97^c^		
**WHtR**	≤ 0.5 (ref)	1			1		
	> 0.5	1.26	0.24–6.73	0.7897	4.71^c^	0.17–134.82	0.3646

^a^ waist-to-height ratio tertiles: Male: ≤ 0.55 T1, > 0.55- ≤ 0.59 T2, > 0.59 T3; Female: ≤ 0.56 T1, > 0.56- ≤ 0.6 T2, > 0.6 T3

(ref)-reference category

^b^ Male ≤ 0.59 v > 0.59, Female ≤ 0.6 v > 0.6, & ≤ 0.50 v > 0.50; all models adjusted for age, race/ethnicity, parents education, occupation

^c^ Firth Bias Corrected Logistic regression

As the WHtR tertile cut points were similar in both sexes, we also examined the association between common WHtR tertile cut points and HOMA-IR and liver enzymes. The results suggested a similar pattern to sex-specific results. (Supplemental Table 2).

## Discussion

The study's primary finding confirmed our hypothesis and suggested that higher WHtR is associated with an unfavorable cardiometabolic profile, specifically IR and elevated liver biomarkers. The odds ratio magnitude with WHTR tertiles was more substantial in female than male children except for NASPGHAN ALT and AST.

Within this cohort of 7–12-year-old children with a BMI ≥ 85^th^ percentile for age and sex, children in the upper tertile for WHtR had almost 1.83 -5.68-fold higher odds of elevated liver enzyme levels and IR than children in the lowest tertile in both sexes. These results are consistent with previous studies in adults [21, 23, 33] analyzing various WHtR thresholds predictive of higher cardiometabolic risk, adding to the research on anthropometric predictor value and cardiometabolic risk in pediatric populations. Ashwell and Hsieh [13] suggested dichotomized optimal WHtR cut point of 0.5 for both children and adults among different ethnic groups between both sexes. While Khoury et al. [25, 44] used arbitrary cut points < 0.5, > 0.5 to < 0.6, ≥ 0.6 in combination with BMI, showed higher WHtR categories were significant risk factors for lipid and cardiometabolic markers in children with obesity. Another study [45] used 0.512 as the WHtR cut point, ignoring the child’s sex, and concluded there is little difference between BMI and WHtR but preferred WHtR in identifying children with adverse cardiovascular disease (CVD) risk factors. A recent meta-analysis [18] of diagnostic studies assessing the WHtR cut-off value suggested an optimal practical cut point of 0.5; however, this was not replicated in our cohort. A difference between the study cohorts may be their makeup; ours is composed predominantly of Hispanic and Black children with a BMI ≥ 85^th^ percentile for age and sex. This cut-off > 0.6 has been suggested by other studies [25, 42, 43] that showed a similar robust association with cardiometabolic risk and metabolic syndrome in children with obesity.

ALT and AST are widely used as noninvasive screening tools for NAFLD and non-alcoholic steatohepatitis (NASH) in the pediatric population [46]. Although ALT is suggested as currently the best inexpensive screener of NAFLD in children [36], it has limitations such that there is no consensus on ALT normal values, and not all ALT-positive screening will have liver disease, leading to inconsistencies [47]. While proportional relationships between the liver enzymes and varying WHtR thresholds have been established [13, 21, 33], this was not replicated in our cohort, except for ALT (≥ 30). ALT) was nearly threefold higher in female children in WHtR T3 compared to T1. When ALT was assessed using sex-specific NASPGHAN ALT criteria, there was a stronger association with WHtR in both sexes; T3 WHtR compared to the T1 WHtR category.

Obesity screening programs could be incorporated into pediatric settings such as schools and conducted with protocols similar to those used in school FitnessGram and other obesity evaluations [48, 49]. Using the WHtR as a screening tool in schools and public health settings could quickly identify high-risk children who should be referred for further assessment. A population-based screening should be conducted in safe, confidential spaces to minimize stigmatizing children with overweight and obesity. Our study suggests that WHtR could be helpful in identifying children with an unhealthy phenotype of obesity.

Strengths of this study include the scientific rigor of data collection, the availability of a database with an adequate sample size to test the hypotheses, the interface with safety-net primary care, the availability of relevant cardiometabolic biomarkers, and diverse ethnic/racial distributions of the participants.

Study limitations include a single measure of anthropometric and biochemical measures obtained at baseline, hence, the cross-sectional design. Consistent with the parent study protocol criteria, all the children in the trial were overweight or obese, so we did not have a normal weight control group to perform a comparative analysis. Although we had a relatively large sample size, we may not have had adequate statistical power to assess sex-specific differences. The population's demographic characteristics (majority of parents/guardians identified as Hispanic and were born outside of the continental United States) and setting (pediatric safety-net primary care) potentially limit the generalizability of our results.

## Conclusion

Assessing WHtR may prove to be an efficient and quick screening method to identify children with overweight and obesity who are at elevated risk for cardiometabolic disorders, particularly those who have IR and elevated liver biomarkers. The approach minimizes the stigma or social disparities associated with obesity. This screening method is feasible for use in schools and other pediatric environments, such as fitness grams and evaluations.

### Supplementary Information

**Additional file 1:** **Supplemental Table 1.** Distribution of Different Waist-to-Height Ratio (WHtR) categories by Sex.

## Data Availability

The datasets generated during and/or analyzed during the current study are not publicly available. Questions about access can be directed to the Study PI’s Drs. Wylie-Rosett (5R18DK075981) and Lichtenstein (5R01HL101236).

## Abbreviations

WHtR: Waist-to-height ratio
HOMA-IR: Homeostatic Model Assessment for Insulin Resistance
AST: Aspartate aminotransferase
ALT: Alanine aminotransferase
TG: Triglyceride,
TC: Total cholesterol
LDL: Low-Density Lipoprotein
HDL-C: High-Density Lipoprotein Cholesterol
SD: Standard Deviation
IR: Insulin Resistance
NAFLD: Non-Alcoholic Fatty Liver Disease
NASH: Non-Alcoholic Steatohepatitis
SGOT: Serum Glutamic-Oxaloacetic Transaminase
SGPT: Serum Glutamate Pyruvate Transaminase
NASPGHAN: North American Society For Pediatric Gastroenterology, Hepatology & Nutrition
aOR: Adjusted Odds Ratio
BMI: Body Mass Index
CVD: Cardiovascular disease
IRB: Institutional Review Board
