# Supplementary material for: Waist-to-height ratio associated cardiometabolic risk phenotype in children with overweight/obesity
Source: BMC Public Health. 2023 Aug 15;23:1549. doi: 10.1186/s12889-023-16418-9 (PMC10426079; doi:10.1186/s12889-023-16418-9)
Supplement: Supplementary file 1 — Additional file 1: Supplemental Table 1. Distribution of Different Waist-to-Height Ratio (WHtR) categories by Sex. [file 12889_2023_16418_MOESM1_ESM.docx]

**Supplemental Table 1: Distribution of Different Waist-to-Height Ratio (WHtR) categories by Sex**

| **Waist-to-Height Ratio (WHtR)** | **Sex** | | **P-value** |
| --- | --- | --- | --- |
|  | **Male**  **(n = 175)** | **Female**  **(n = 185)** |  |
| **Sex-specific Tertile*,**  n (%) |  |  | 1.00 |
| T1 | 58 (33.1) | 61 (33.0) |  |
| T2 | 59 (33.7) | 62 (33.5) |  |
| T3 | 58 (33.1) | 62 (33.5) |  |
| **Sex-specific WHtR Binary cut** |  |  | 0.98 |
| M: ≤0.59; F: ≤0.60, | 109 (62.3) | 115 (62.2) |  |
| M: > 0.59; F: >0.60 | 66 (37.7) | 70 (37.8) |  |
| **Common WHtR Binary cut (0.55)** |  |  | 0.25 |
| ≤0.55 | 54 (30.9) | 47 (25.4) |  |
| >0.55 | 121 (69.1) | 138 (74.6) |  |
| **Common WHtR Binary cut (0.50)** |  |  | 0.71 |
| ≤0.50 | 9 (5.1) | 8 (4.3) |  |
| >0.50 | 166 (94.9) | 177 (95.7) |  |

* Male: ≤0.55 T1, >0.55 - ≤0.59 T2, >0.59 T3; Female: ≤0.56 T1, >0.56-≤0.6 T2, >0.6 T3.

**Supplemental Table 2: Odds Ratio, 95% confidence interval, and P-value from multivariable logistic regression for outcomes HOMA-IR, AST, and ALT**

| **Variable** | | **HOMA-IR** | | | **ALT(SGPT)**  **<30, ≥30** | | | **ALT(SGPT)**  **Male: ≤26, > 26/ Female: ≤22, > 22** | | | **AST(SGOT)**  **<36, ≥36** | | |
| --- | --- | --- | --- | --- | --- | --- | --- | --- | --- | --- | --- | --- | --- |
|  |  | **aOR** | **95% CI** | **P**  **value** | **aOR** | **95% CI** | **P**  **value** | **aOR** | **95% CI** | **P**  **value** | **aOR** | **95% CI** | **P-value** |
| **WHtR^#^** | T1 (ref)  T2  T3 | 1  **2.76**  **4.85** | **1.46-5.23**  **2.53-9.28** | **0.0018**  **<0.0001** | 1  1.55  **2.33** | 0.76-3.14  **1.20-4.55** | 0.2254  **0.0130** | **1**  **1.73**  **2.50** | **0.99-3.00**  **1.46-4.28** | **0.0543**  **0.0009** | 1  1.04  1.49 | 0.52-2.09  0.77-2.86 | 0.9162  0.2368 |
| **WHtR^#^*** | T1 (ref)  T2  T3 | 1  **2.87**  **4.82** | **1.57-5.23**  **2.64-8.81** | **0.0006**  **<0.0001** | - |  |  | - |  |  | - |  |  |
|  | | | | | | | | | | | | | |
| **WHtR^§^** | M: ≤0.59; F: ≤0.60 (ref)  M: > 0.59; F: >0.60 | 1  **3.26** | **1.89-5.63** | **<0.0001** | 1  **1.84** | **1.07-3.16** | **0.0267** | **1**  **2.01** | **1.29-3.14** | **0.0021** | 1  1.38 | 0.79-2.42 | 0.2631 |
| **WHtR^§*^** | M: ≤0.59; F: ≤0.60 (ref)  M: > 0.59; F: >0.60 | 1  **3.46** | **2.07-5.79** | **<0.0001** | - |  |  | - |  |  | - |  |  |
|  | | | | | | | | | | | | | |
| **WHtR** | ≤0.55 (ref)  > 0.55 | 1  **3.31** | **1.83-5.99** | **<0.0001** | **1**  **1.84** | **0.96-3.51** | **0.0664** | 1  **2.03** | **1.22-3.39** | **0.0068** | 1  1.28 | 0.69-2.38 | 0.4314 |
| **WHtR^*^** | ≤0.55 (ref)  > 0.55 | **1**  **3.47** | **2.00-6.02** | **<0.0001** | - |  |  | - |  |  | - |  |  |
|  | | | | | | | | | | | | | |
| **WHtR** | ≤0.5 (ref)  > 0.5 | 1  1.75 | 0.53-5.80 | 0.3605 | 1  3.79 | 0.48-29.89 | 0.2056 | **1.00**  **2.98** | **0.82-10.83** | **0.0979** | 1  1.85 | 0.39-8.78 | 0.4367 |
| **WHtR^*^** | ≤0.5 (ref)  > 0.5 | 1  2.29 | 0.71-7.43 | 0.1666 | - |  |  | - |  |  | - |  |  |

# waist-to-height ratio tertiles: male: ≤0.55 T1, >0.55-≤0.59 T2, >0.59 T3; female: ≤0.56 T1, >0.56-≤0.6 T2, >0.6 T3; (ref)-reference category, § Male ≤ 0.59 v >0.59, Female ≤0.6 v>0.6, & ≤ 0.50 v >0.50; all models adjusted for age, race/ethnicity, parents education, occupation, HOMA-IR models were additionally adjusted for tanner stage; * multiple imputations estimates
